# Influence of the Interface Composition to the Superconductivity of Ti/PdAu Films

**DOI:** 10.3390/nano11010039

**Published:** 2020-12-25

**Authors:** Xiaolong Xu, Mauro Rajteri, Jinjin Li, Shuo Zhang, Jian Chen, Eugenio Monticone, Qing Zhong, Huifang Gao, Wei Li, Xu Li, Qi Li, Yuan Zhong, Wenhui Cao, Shijian Wang, Ying Gao, Zheng Liu, Xueshen Wang

**Affiliations:** 1 National Institute of Metrology (NIM), Beijing 100029, China; xiaolong.xu@nim.ac.cn (X.X.); chenjian@nim.ac.cn (J.C.); zhongq@nim.ac.cn (Q.Z.); gaohf@nim.ac.cn (H.G.); liwei@nim.ac.cn (W.L.); li-xu@nim.ac.cn (X.L.); liqi@nim.ac.cn (Q.L.); zhongyuan@nim.ac.cn (Y.Z.); caowh@nim.ac.cn (W.C.); wangshj@nim.ac.cn (S.W.); gaoying2018@nim.ac.cn (Y.G.); lzheng@nim.ac.cn (Z.L.); 2 Istituto Nazionale di Ricerca Metrologica (INRiM), 10135 Torino, Italy; m.rajteri@inrim.it (M.R.); e.monticone@inrim.it (E.M.); 3 Center for Transformative Science, ShanghaiTech University, Shanghai 201210, China; shuozhang@shanghaitech.edu.cn

**Keywords:** Ti/PdAu, proximity effect, interfere, composition, annealing, superconductivity

## Abstract

In this work, the interface composition of the superconducting Ti/PdAu bilayer is tuned by an annealing process in N_2_ from 100 to 500 °C to control the superconducting transition temperature (*T*_c_). This Ti-PdAu composition layer is characterized with a high-resolution transmission electron microscopy (HRTEM) and energy-dispersive spectrometer (EDS) to show the infiltration process. The surface topography, electrical, and cryogenic properties are also shown. The inter-infiltration of Ti and PdAu induced by the thermal treatments generates an intermixed layer at the interface of the bilayer film. Due to the enforced proximity effect by the annealing process, the *T*_c_ of Ti (55 nm)/PdAu (60 nm) bilayer thin films is tuned from an initial value of 243 to 111 mK which is a temperature that is suitable for the application as the function unit of a superconducting transition edge sensor.

## 1. Introduction

Superconducting transition-edge sensors (TES) [1] have been widely used in the infrared-visible region [2], and for X-ray [3] and γ-ray [4] detection with the superiority of photon-number and energy resolving capability, high quantum efficiency, and negligible dark-count rate. TES usually works on the sharp transition edge between the superconducting and normal state of superconducting films, and the energy resolution (Δ*E*) in the strong electro-thermal feedback is expressed as follows [5]:

(1) $$\Delta E=\sqrt{\frac{4kT_{c}^{2}C\sqrt{n/2}}{\alpha }}\propto T_{c}^{\frac{3}{2}}$$

where *k* is the Boltzmann constant, *n* is an exponent factor of thermal conductance, *C* and *α* are the heat capacity and thermal sensitivity of a superconducting thin film which acts the key function unit of a TES. From (1), we can see that the *T*_c_ should be as low as possible to obtain a better Δ*E* [6].

Superconducting Ti films have been widely used especially for optical TES. The *T*_c_ of pure Ti is from 360 to 500 mK [7,8,9,10,11] as the thicknesses changes. However, for a better Δ*E*, the *T*_c_ should be ≈ 100 mK [12,13,14,15,16,17]. The tunable range of *T*_c_ of a pure Ti film is limited. The common method to control the *T*_c_ is exploiting the proximity effect [18,19,20,21] between a superconducting and a normal metal film. According to the Usadel theory [19,22], the *T*_c_ of a bilayer film composed by a superconducting film layer and a normal metal film layer is expressed as:

(2) $$T_{c}=\;T_{c0}{\left[\frac{d_{s}}{d_{0}}\frac{1}{1.13\left(1+\frac{1}{\beta }\right)}\frac{1}{t}\right]}^{\beta }\;\frac{1}{d_{0}}=\frac{\pi }{2}{kT}_{c0}\lambda_{F}^{2}n_{s}\;\beta =d_{n}n_{n}/d_{s}n_{s}$$

Here *d*_n_ and *d*_s_ are the thickness of the normal metal film and the superconducting metal film, *n*_n_ and *n*_s_ are the density of states for the respective materials, *T*_c0_ is the *T*_c_ of the superconducting metal film, *λ*_F_ is the Fermi wavelength of the normal metal, and *t* is the unitless modified parameter to describe the transmission through the interface of the bilayer film. From (2), we can see that there are two methods to tune the *T*_c_ of a pure Ti film: one is changing the thickness ratio *d*_n_/*d*_s_, the other is tuning the interface property *t*. The interface status plays a key role to influence the *T*_c_. Au is usually used as the normal metal for Ti [23,24,25,26,27,28,29,30,31,32]. However, nanometer Au films are not stable, and the surface roughness becomes larger as time goes by. Moreover, the baking temperature obviously affected the *T*_c_ of Ti/Au films even below 100 °C [27].

In this paper, a PdAu alloy film, which is more stable than a pure Au film, is used as the normal metal film to tune the *T*_c_ of Ti films. An annealing process in N_2_ from 100 to 500 °C is performed. The inter-infiltration of Ti and PdAu induced by the thermal treatments enforces the proximity effect and tunes the *T*_c_ from an initial value of 243 to 111 mK. From the results of the high-resolution transmission electron microscopy (HRTEM), energy-dispersive spectrometer (EDS), X-ray diffraction (XRD), surface topography, and electrical characterization, to obviously tune the *T*_c_ (>10%), the annealing temperature should be above 100 °C, which is beneficial for the nanofabrication process and application of TES using Ti/PdAu films.

## 2. Materials and Methods

A commercial 3-inch monocrystalline silicon substrate with a 500 nm low-pressure chemical vapor deposition (LPCVD) SiN_x_ layer is cleaned with acetone, isopropyl alcohol, ethanol, de-ionized water, and then dried with N_2_, in sequence. A Ti (55 nm)/PdAu (60 nm) bilayer thin film is deposited on it using an ultrahigh vacuum confocal DC magnetron sputtering system (Sky technology development ltd., Shenyang, China). The base pressure of the main chamber is ≈10^−6^ Pa. The deposition rates are 1.1 Å/s for the Ti and 7.4 Å/s for the PdAu layer. During the deposition process, the substrate temperature is kept at 20 °C by a circulating water cooler. The films are cut into slices of 5 mm × 10 mm for the following annealing process.

**Figure 1:**
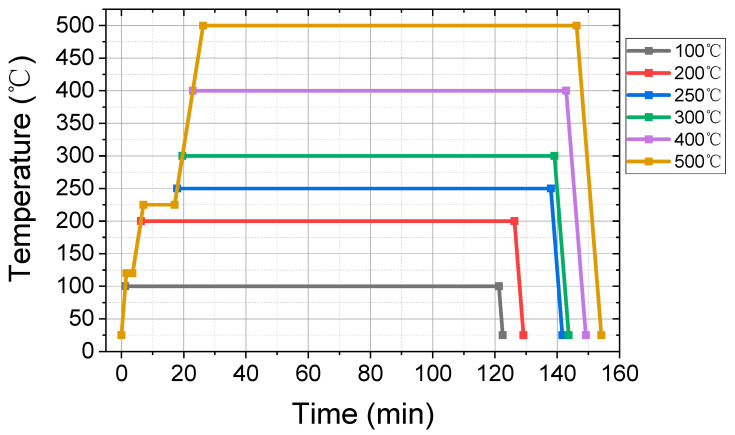
The annealing progress of Ti (55 nm)/PdAu (60 nm) films.

The annealing process is performed in high-purity N_2_ as the protection atmosphere with a programmable UniTemp GmbH RTP-100 oven (Universal Temperature Processes, Pfaffenhofen, Germany). Six slices of 5 mm × 10 mm samples from the same 3-inch film is used to perform the annealing process with the temperature ranges from 100 to 500 °C respectively, as shown in Figure 1. The films are firstly heated to 120 °C with a rate of 1 °C/s and kept for 2 min to remove the humidity. Above 120 °C, the heating rate is set to 0.5 °C/s, and the temperature is kept at 225 °C for 10 min to make the potential organic matter gasified out. The maximum temperatures are held for 2 h to realize sufficient annealing. Finally, the cooling rate is set as −1 °C/s to room temperature.

## 3. Results

### 3.1. Morphology of Interface Composition

**Figure 2:**
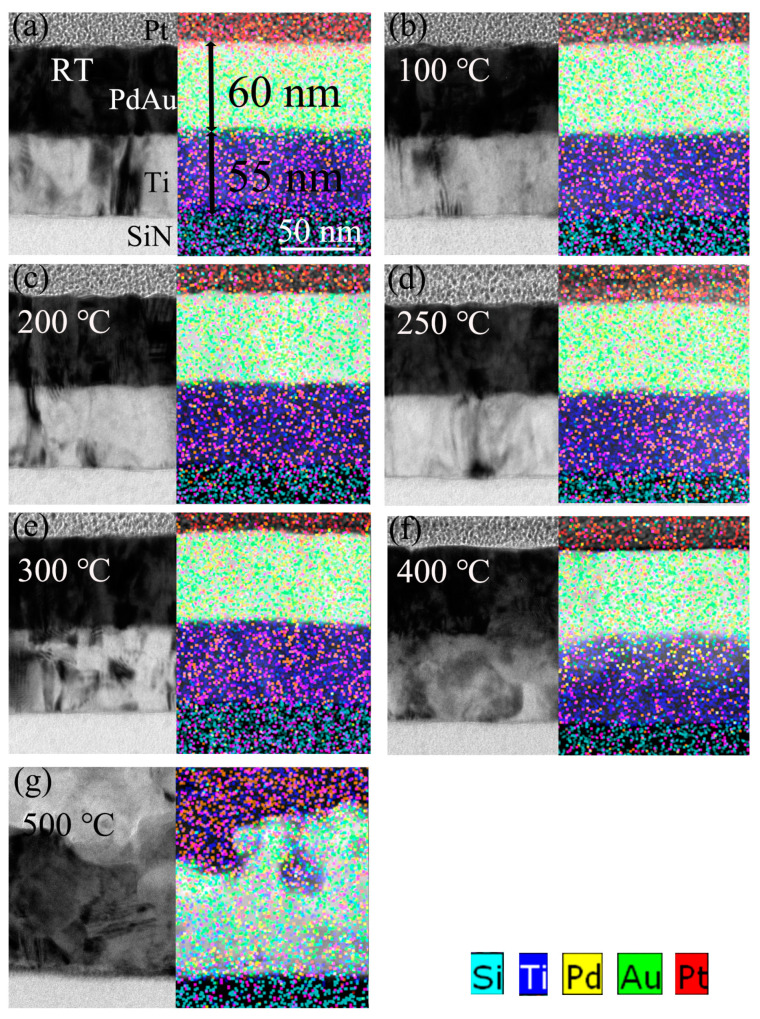
SEM and EDS of the cross-section of the Ti/PdAu bilayer films, annealing temperature at (**a**) RT, (**b**) 100 °C, (**c**) 200 °C, (**d**) 250 °C, (**e**) 300 °C, (**f**) 400 °C, (**g**) 500 °C.

After the annealing process, the cross-section of the bilayer Ti/PdAu films are fabricated by a focus ion beam milling method with a Pt layer as the protective layer. Then a 55 nm Ti layer and 60 nm PdAu layer are clearly shown in the SEM and EDS images of Figure 2. The interface is clear below 300 °C, and the intermixed layer is not obviously shown in Figure 2a–d, presumably because the extent of inter-diffusion is not sufficient for a FEI Helios NanoLab G3 SEM characterization (FEI, Hillsboro, OR, USA). From the EDS analysis (FEI, Hillsboro, OR, USA), when the annealing temperature is lower than 300 °C, the atoms diffuse only several nanometers to ≈ 10 nm at the interface and slightly deeper and deeper when the annealing temperature increases.

However, when the annealing temperature is 400 °C, the interface shows obviously an atomic inter-diffusion blurring the interface, as shown in Figure 2f. The two layers start to mix, and a Ti-PdAu intermixed layer with a thickness around 40 nm generates. Moreover, when the annealing temperature rises to 500 °C, as shown in Figure 2g, atomic diffusion is enhanced to the extent that Ti diffuses uniformly throughout the whole structure and PdAu diffuses to the bottom layer. The atomic diffusion at the interface will enforce the proximity effect and tune the *T*_c_ of the Ti/PdAu films [22].

**Figure 3:**
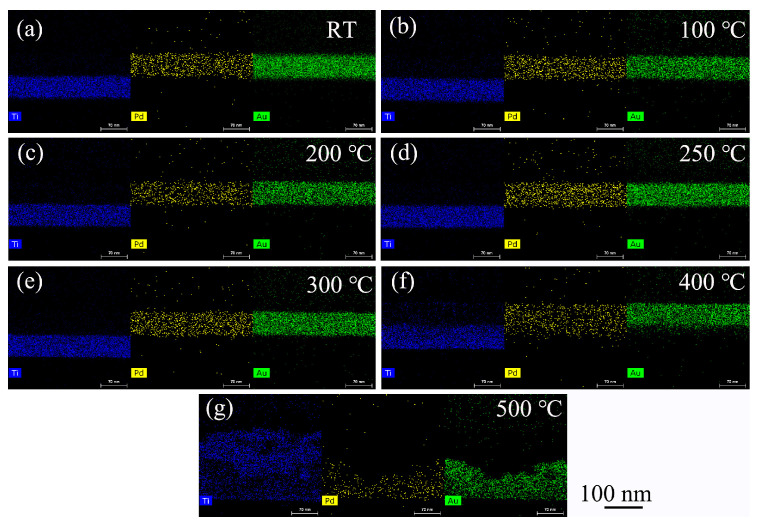
Element surface distribution of the cross-section of Ti/PdAu bilayer films, annealing temperature at (**a**) RT, (**b**) 100 °C, (**c**) 200 °C, (**d**) 250 °C, (**e**) 300 °C, (**f**) 400 °C, (**g**) 500 °C.

Figure 3 shows the Ti, Pd, and Au surface elements distribution at the cross-section of the Ti/PdAu layer and demonstrates the diffusion process of Ti and PdAu at the interface. The element distribution of the bilayer structure is separated at the Z-axis to Ti, PdAu bilayers. In Figure 3a, the Pd and Au elements stay at the same altitude, which means PdAu is the alloy that stays at same layer. The Ti layer is just below the PdAu layer. When the annealing temperature increases, the Ti, Pd, and Au atoms cross the interface and start the diffusion process. When the annealing temperature rises to 400 °C, as shown in Figure 2f, there is a 40 nm thick layer in which Ti, Pd and Au atoms are intermixed. When the annealing temperature increases to 500 °C, as shown in Figure 2g, Ti atoms are distributed throughout the original area of the Ti/PdAu layers and Pd as well as Au atoms diffuse into the lower layer. The whole bilayer area is converted into a Ti-Pd-Au intermixed layer.

**Figure 4:**
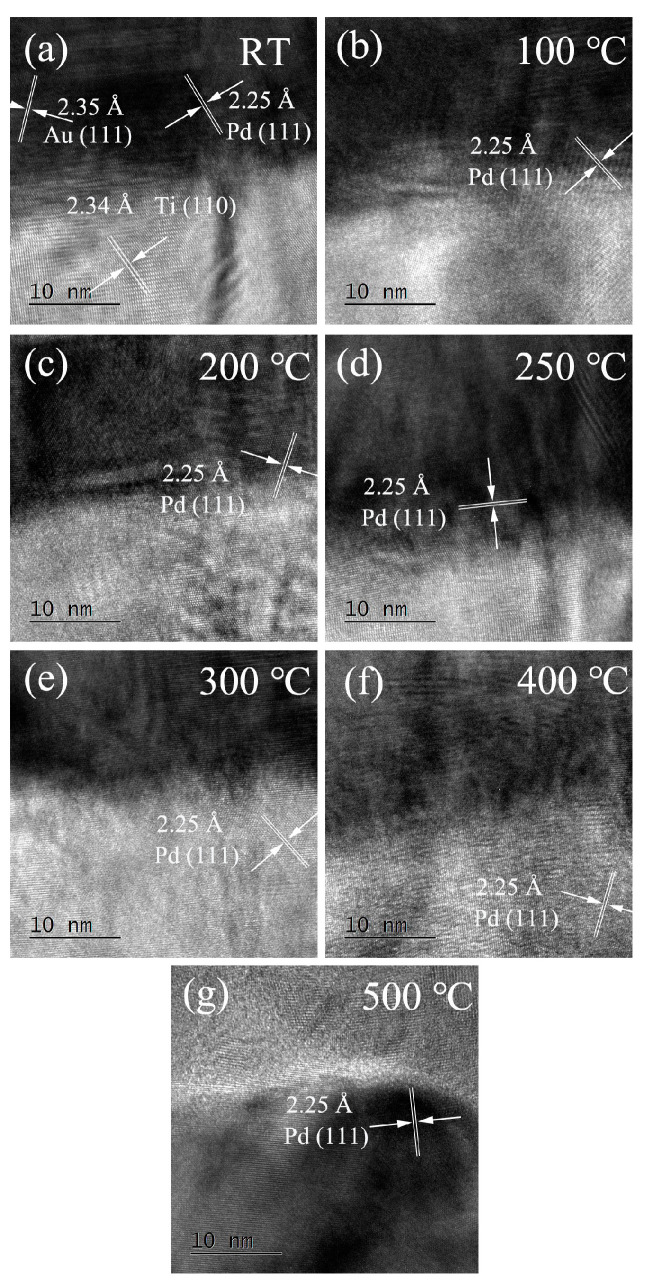
HRTEM characterization of Ti/PdAu interface of the Ti/PdAu bilayer thin film, annealing temperature at (**a**) RT, (**b**) 100 °C, (**c**) 200 °C, (**d**) 250 °C, (**e**) 300 °C, (**f**) 400 °C, (**g**) 500 °C.

The Ti/PdAu interface is also characterized by FEI Tecnai F20 HRTEM (FEI, Hillsboro, OR, USA), as shown in Figure 4. The spacing (bright part) between the (110) lattice planes of the cubic Ti crystal is 2.34 Å, and that of the (111) planes of cubic Au and Pd (dark part) are 2.35 Å and 2.25 Å separately. Considering that the lattice planes of Ti (110) and Au (111) are quite similar, Pd (111) is selected as the characteristic factor to identify atomic diffusion across the interface. Pd (111) lattice planes can be identified only near the interface when the annealing temperature is low. When the annealing temperature rises above 300 °C, the Pd atom crosses the interface and diffuse into the Ti layer. For 500 °C, the Ti and PdAu phase mix, redistribute, and enrich in inversion. The HRTEM results shows the consistency with the EDS characterization.

**Figure 5:**
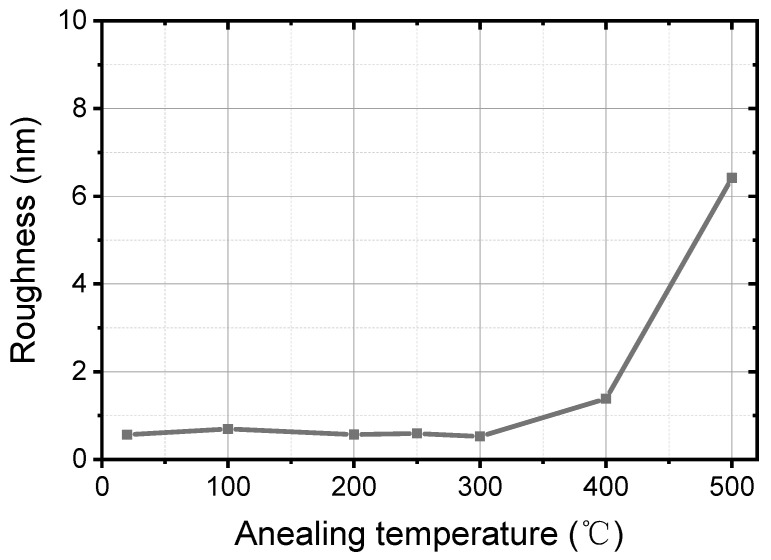
Roughness of Ti/PdAu bilayer thin film vs. annealing temperature.

The roughness of the annealed Ti/PdAu thin films is measured on a scan size of 2 µm × 2 µm using a Veeco Dimension Icon system (Veeco, New York, NY, USA). The result of roughness *R*_q_ (root mean surface squared roughness) is plotted vs. annealing temperature as shown in Figure 5. When the annealing temperature is below 300 °C, the *R*_q_ is around 0.6 nm, which is similar or better than in previous work [7,12,15,20,30], as shown in Table 1. With good morphological property (lower roughness), the thin films present better performance in proximity effect and robust process compatibility in further TES fabrication with the multilayer readout wiring.

*R*_q_ increases to 1.4 nm at 400 °C. Afterwards, an abrupt jump to 6.4 nm (i.e., 1 order of magnitude) is observed as the annealing temperature reaches 500 °C. It is mainly attributed to the atom diffusion, which also could be obvious in Figure 3g.

### 3.2. Structure and Phase

**Figure 6:**
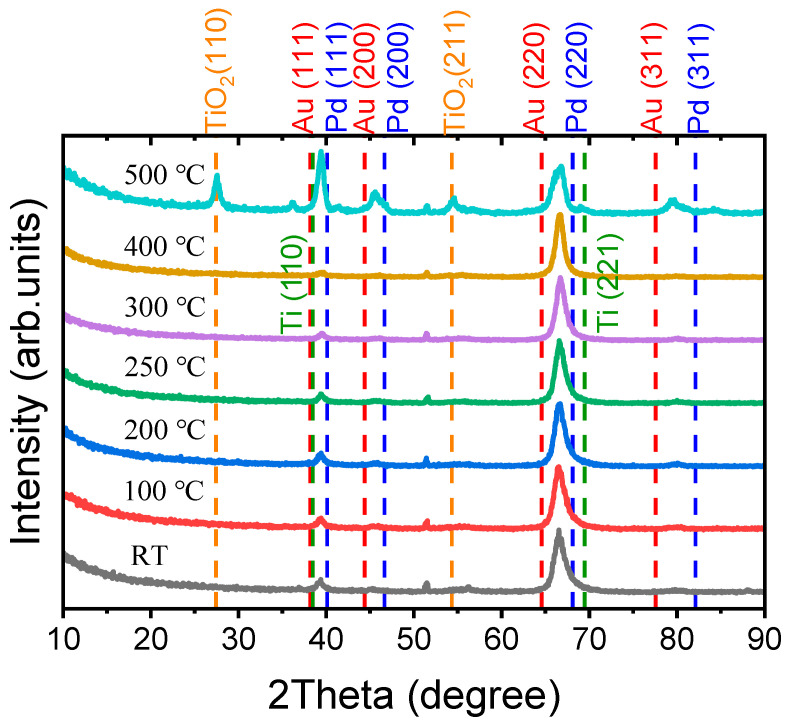
XRD pattern of annealed Ti/PdAu bilayer thin films.

The X-ray diffraction (XRD) pattern of the films was performed using a Panalytical X’Pert PRO MPD diffractometer (Cu λKα = 1.541874 Å) (Malvern Panalytical Ltd, Malvern, United Kingdom) with the incidence angle fixed at 0.5°, and the *2θ* angle ranged from 10° to 90° with a step of 0.05°, as shown in Figure 6. Two strong preferential orientation of PdAu alloy (Gold, JCPDS card # 04-0784 and Palladium, JCPDS card # 46-1043) peaks are clearly recorded at *2θ* = 39.52° and 66.82°, which represent the (111) and (220) planes of the PdAu alloy phase. In addition, two weaker diffraction signals at *2θ* = 45.73° and 80.26° correspond to the PdAu alloy (200) and (311) planes, respectively [33,34,35,36]. The peak at *2θ* = 69.48° is mainly due to Ti (211) planes (Titanium, JCPDS card # 44-1288), and another Ti (110) plane appears at *2θ* = 38.48°, which is merged under the high intensity of the PdAu (111) plane. PdAu alloy has heavy density, which could easily scatter the X-ray from the copper cathode, and the small incidence angle (0.5°) increases the path length of the X-ray. These effects make the signal from the Ti layer weak. When the annealing temperature is 500 °C, two strong peaks of the rutile TiO_2_ (110) phase (TiO_2_, JCPDS card #21-1276) appear at *2θ* = 27.45°, and the rutile TiO_2_ (211) phase appears at 2θ = 54.32°. Thermal oxidation of the Ti at 500 °C causes the appearance of the TiO_2_ peaks.

### 3.3. Electrical Property

**Figure 7:**
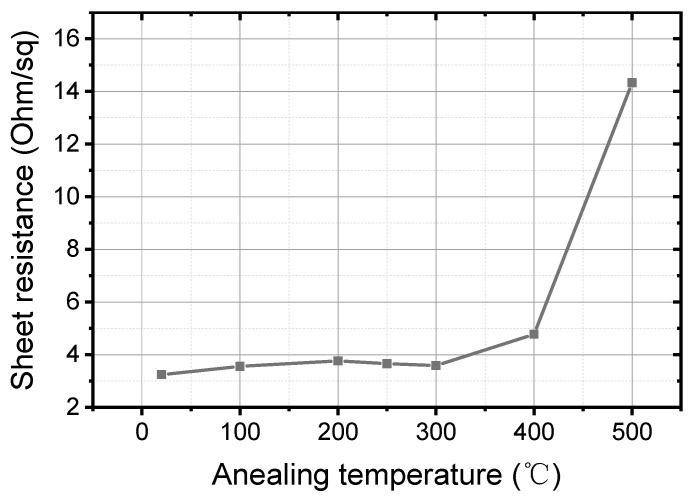
Sheet resistance vs. annealing temperature of Ti/PdAu bilayer thin film.

The sheet resistance *R*_□_ of Ti/PdAu bilayer films is an important factor influenced by the annealing temperature and affects the sensitivity of the voltage biased TES. *R*_□_ is determined by mapping measurements with a CDE Resmap 178 system (Creative Design Engineering Inc., Cupertino, CA, USA) based on the van der Pauw method. Figure 7 shows the *R*_□_ vs. temperatures. When the annealing temperature is lower than 300 °C, the *R*_□_ is around 3.5 Ω/□. Above 300 °C, *R*_□_ significantly increases because of the thermal oxidation of Ti and interface composition.

### 3.4. Cryogenic Property

**Figure 8:**
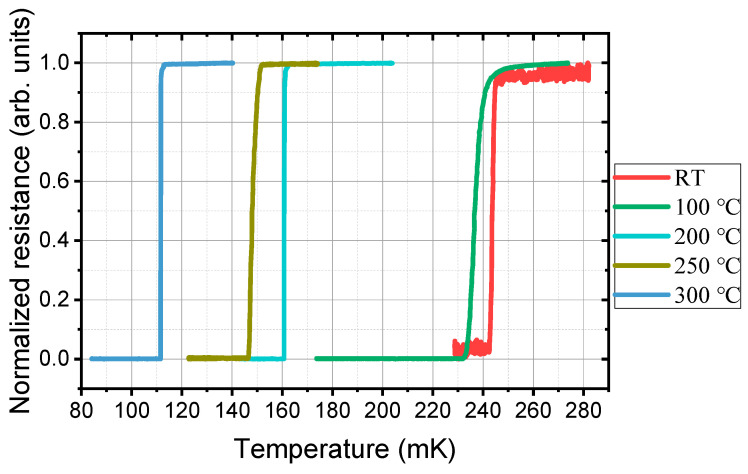
The effect of annealed treatment on the *T*_c_ of Ti/PdAu bilayer films.

Figure 8 shows the resistance temperature curve of the annealed Ti/PdAu films measured in an adiabatic demagnetization refrigerator (ADR) system (High Precision Devices, Inc., Boulder, CO, USA). After the annealing at 100 °C, the *T*_c_ is 236.5 mK, which is slightly lower than the unannealed films (243.5 mK). As the annealing temperature increases, the *T*_c_ gradually decreases from 160.4 mK (200 °C) to 147.4 mK (250 °C), and then to 111.5 mK (300 °C). Above 400 °C, the temperature maybe too high as shown in the HRTEM and SEM characterization, the Ti/PdAu films do not show superconducting transition down to 30 mK. For the TES application equipped in a dilution refrigerator or ADR, *T*_c_ ≈ 100 mK is suitable. Therefore, the annealing process at 300 °C is applicable.

## 4. Conclusions

A 60 nm PdAu film is deposited on the top surface of 55 nm Ti to tune the *T*_c_ of the Ti film. An annealing process is performed to modify the proximity effect. After annealing, Ti and PdAu atoms recombine at the Ti/PdAu interface to form an intermixed layer. Due to the intermixed layer, the *T*_c_ of the Ti/PdAu bilayer film is successfully tuned from 243 mK to the ideal 111 mK, which is optimal for TES applications. The *T*_c_ could be controlled by the interface property without changing the thickness ratio between the superconducting and normal metal. The annealing temperature should be below 400 °C. Otherwise, the bilayer film will show a normal metal state even at the temperature down to 50 mK. The Ti-PdAu intermixed layer at the interface is characterized by SEM, EDS and HRTEM to analyze the mechanism of *T*_c_ adjustment. As a new superconducting/normal film combination, annealed Ti/PdAu bilayer films have shown great potential for TES applications.

## Figures and Tables

**Table 1:** Roughness of the superconductive thin film used in TES.

	Ref. [7]	Ref. [15]	Ref. [12]	Ref. [20]	Ref. [30]	This Work
Superconductive materials	Ti	Mo	Mo/Cu	Ti/Au	Ti/Au	Ti/PdAu
Thickness	100 nm	200 nm	72 nm/95 nm	40 nm/70 nm	100 nm/20 nm	55 nm/60 nm
Roughness *R*_q_	1.5 nm	1.7 nm	0.75 nm	4.5 nm	0.4 nm	0.6 nm

## References

[1] Lita A.E., Miller A.J., Nam S.W. (2008). Counting near-infrared single-photons with 95% efficiency. Opt. Express.

[2] Taralli E., Lolli L., Portesi C., Monticone E., Rajteri M., Wang T.-S., Chen J.-K., Zhou X. (2015). Reduced Active Area in Transition-Edge Sensors for Visible-NIR Photon Detection: A Comparison of Experimental Data and Two-Fluid Model. IEEE Trans. Appl. Supercond..

[3] Jaeckel F.T., Ambarish C.V., Christensen N., Gruenke R., Hu L., McCammon D., McPheron M., Meyer M., Nelms K.L., Roy A. (2019). Energy Calibration of High-Resolution X-Ray TES Microcalorimeters with 3 eV Optical Photons. IEEE Trans. Appl. Supercond..

[4] Jaeckel F.T., Le L.N., Martin K.W., Boyd S.T.P. (2013). Development of a Precision Scanning Optical Pulser for Low-Temperature Particle Detectors. IEEE Trans. Appl. Supercond..

[5] Enss C., Enss C. (2005). Cryogenic Particle Detection.

[6] Lolli L., Taralli E., Rajteri M., Numata T., Fukuda D. (2013). Characterization of Optical Fast Transition-Edge Sensors With Optimized Fiber Coupling. IEEE Trans. Appl. Supercond..

[7] Xu X., Li J., Wang X., Zhong Q., Zhong Y., Cao W., Li W., Chen J., Zhao Z., Gao Y. Investigation of Superconducting Titanium films for Transition Edge Sensors. Proceedings of the 2020 Conference on Precision Electromagnetic Measurements (CPEM).

[8] Bonetti J.A., Day P.K., Kenyon M., Kuo C.L., Turner A., LeDuc H.G., Bock J.J. (2009). Characterization of antenna-coupled tes bolometers for the spider experiment. IEEE Trans. Appl. Supercond..

[9] Portesi C., Taralli E., Lolli L., Rajteri M., Monticone E. (2015). Fabrication and Characterization of Fast TESs With Small Area for Single Photon Counting. IEEE Trans. Appl. Supercond..

[10] Vaccaro D., Siri B., Baldini A.M., Biasotti M., Cei F., Ceriale V., De Gerone M., Galli L., Gallucci G., Gatti F. (2018). Tuning the TC of Titanium Thin Films for Transition-Edge Sensors by Annealing in Argon. J. Low Temp. Phys..

[11] Fukuda D., Fujii G., Numata T., Amemiya K., Yoshizawa A., Tsuchida H., Fujino H., Ishii H., Itatani T., Inoue S. (2011). Titanium-based transition-edge photon number resolving detector with 98% detection efficiency with index-matched small-gap fiber coupling. Opt. Express.

[12] Jaeckel F.T., Kripps K.L., Morgan K.M., Zhang S., McCammon D. (2016). Fabrication of Superconducting Mo/Cu Bilayers Using Ion-Beam-Assisted e-Beam Evaporation. J. Low Temp. Phys..

[13] Jaeckel F.T., Kripps K.L., McCammon D., Wulf D., Zhang S., Zhou Y. (2017). Effects of Uniaxial Stress on Mo and Mo/Cu Bilayer Superconducting Transitions. IEEE Trans. Appl. Supercond..

[14] Parra-Borderias M., Fernandez-Martinez I., Fabrega L., Camon A., Gil O., Costa-Kramer J.L., Gonzalez-Arrabal R., Sese J., Bueno J., Briones F. (2013). Characterization of a Mo/Au thermometer for ATHENA. IEEE Trans. Appl. Supercond..

[15] Morgan K., Jaeckel F.T., Kripps K.L., McCammon D. (2015). Ion-Beam-Assisted Deposition of Mo Thin Films for TES Applications. IEEE Trans. Appl. Supercond..

[16] Lolli L., Taralli E., Portesi C., Rajteri M., Monticone E. (2016). Aluminum–Titanium Bilayer for Near-Infrared Transition Edge Sensors. Sensors.

[17] Taralli E., Portesi C., Rocci R., Rajteri M., Monticone E. (2009). Investigation of Ti/Pd bilayer for single photon detection. IEEE Trans. Appl. Supercond..

[18] Wakeham N.A., Adams J.S., Bandler S.R., Chervenak J.A., Datesman A.M., Eckart M.E., Finkbeiner F.M., Kelley R.L., Kilbourne C.A., Miniussi A.R. (2018). Effects of Normal Metal Features on Superconducting Transition-Edge Sensors. J. Low Temp. Phys..

[19] Hilton G.C., Martinis J.M., Irwin K.D., Bergren N.F., Wollman D.A., Huber M.E., Deiker S., Nam S.W. (2001). Microfabricated transition-edge X-ray detectors. IEEE Trans. Appl. Supercond..

[20] Kuromaru G., Kuwabara K., Miyazaki N., Suzuki S., Hosoya S., Koizumi Y., Ohashi T., Ishisaki Y., Ezoe Y., Yamada S. (2016). Investigation of Surface Roughness Effect on Transition Edge Sensor Microcalorimeters Using Multilayer Readout Wiring. J. Low Temp. Phys..

[21] Sadleir J.E., Smith S.J., Robinson I.K., Finkbeiner F.M., Chervenak J.A., Bandler S.R., Eckart M.E., Kilbourne C.A. (2011). Proximity effects and nonequilibrium superconductivity in transition-edge sensors. Phys. Rev. B Condens. Matter Mater. Phys..

[22] Martinis J.M., Hilton G.C., Irwin K.D., Wollman D.A. (2000). Calculation of TC in a normal-superconductor bilayer using the microscopic-based Usadel theory. Nucl. Instruments Methods Phys. Res. Sect. A Accel. Spectrometers Detect. Assoc. Equip..

[23] Portesi C., Taralli E., Rocci R., Rajteri M., Monticone E. (2008). Fabrication of Au/Ti TESs for optical photon counting. J. Low Temp. Phys..

[24] Lolli L., Taralli E., Rajteri M. (2012). Ti/Au TES to Discriminate Single Photons. J. Low Temp. Phys..

[25] Lolli L., Taralli E., Portesi C., Alberto D., Rajteri M., Monticone E. (2011). Ti/Au transition-edge sensors coupled to single mode optical fibers aligned by Si V-groove. IEEE Trans. Appl. Supercond..

[26] Taralli E., Gottardi L., Nagayoshi K., Ridder M., Visser S., Khosropanah P., Akamatsu H., van der Kuur J., Bruijn M., Gao J.R. (2020). Characterization of High Aspect-Ratio TiAu TES X-ray Microcalorimeter Array Under AC Bias. J. Low Temp. Phys..

[27] Van Der Heijden N.J., Khosropanah P., Van Der Kuur J., Ridder M.L. (2014). Diffusion behaviour in superconducting Ti/Au bilayers for SAFARI TES detectors. J. Low Temp. Phys..

[28] Taralli E., Pobes C., Khosropanah P., Fabrega L., Camón A., Gottardi L., Nagayoshi K., Ridder M.L., Bruijn M.P., Gao J.R. (2020). AC/DC Characterization of a Ti/Au TES with Au/Bi Absorber for X-ray Detection. J. Low Temp. Phys..

[29] Nagayoshi K., Ridder M.L., Bruijn M.P., Gottardi L., Taralli E., Khosropanah P., Akamatsu H., Visser S., Gao J.R. (2020). Development of a Ti/Au TES Microcalorimeter Array as a Backup Sensor for the Athena/X-IFU Instrument. J. Low Temp. Phys..

[30] Kengo K., Ezoe Y., Kitazawa S., Hayakawa R., Nunomura K., Ohashi T., Ishisaki Y., Yamada S., Hidaka M., Satoh T. (2018). Study of Surface Roughness Effect on Super–Normal Transition of Ti/Au Transition Edge Sensor Calorimeters. J. Low Temp. Phys..

[31] Carter F.W., Ade P.A.R., Ahmed Z., Anderson A.J., Austermann J.E., Avva J.S., Thakur R.B., Bender A.N., Benson B.A., Carlstrom J.E. (2018). Tuning SPT-3G Transition-Edge-Sensor Electrical Properties with a Four-Layer Ti–Au–Ti–Au Thin-Film Stack. J. Low Temp. Phys..

[32] Ridder M.L., Nagayoshi K., Bruijn M.P., Gottardi L., Taralli E., Khosropanah P., Akamatsu H., van der Kuur J., Ravensberg K., Visser S. (2020). Study of TES Detector Transition Curve to Optimize the Pixel Design for Frequency-Division Multiplexing Readout. J. Low Temp. Phys..

[33] Xu J., Guo S., Jia L., Zhang W. (2018). Palygorskite supported AuPd alloy nanoparticles as efficient nano-catalysts for the reduction of nitroarenes and dyes at room temperature. Nanomaterials.

[34] Schmidt T.J., Jusys Z., Gasteiger H.A., Behm R.J., Endruschat U., Boennemann H. (2001). On the CO tolerance of novel colloidal PdAu/carbon electrocatalysts. J. Electroanal. Chem..

[35] Feng Y.Y., Liu Z.H., Xu Y., Wang P., Wang W.H., Kong D.S. (2013). Highly active PdAu alloy catalysts for ethanol electro-oxidation. J. Power Sources.

[36] Geraldes A.N., Da Silva D.F., Pino E.S., Da Silva J.C.M., De Souza R.F.B., Hammer P., Spinacé E.V., Neto A.O., Linardi M., Dos Santos M.C. (2013). Ethanol electro-oxidation in an alkaline medium using Pd/C, Au/C and PdAu/C electrocatalysts prepared by electron beam irradiation. Electrochim. Acta.

